# Treating Metabolic Dysregulation and Senescence by Caloric Restriction: Killing Two Birds with One Stone?

**DOI:** 10.3390/antiox14010099

**Published:** 2025-01-16

**Authors:** Lara Russo, Serena Babboni, Maria Grazia Andreassi, Jalil Daher, Paola Canale, Serena Del Turco, Giuseppina Basta

**Affiliations:** 1 Institute of Clinical Physiology, National Research Council, Via Moruzzi 1, 56124 Pisa, Italy; lararusso@cnr.it (L.R.); serena.babboni@ifc.cnr.it (S.B.); andreas@ifc.cnr.it (M.G.A.); paola.canale@santannapisa.it (P.C.); lapina@ifc.cnr.it (G.B.); 2 Department of Biology, Faculty of Arts and Sciences, University of Balamand, El-Koura 100, Lebanon; jalil.daher@balamand.edu.lb

**Keywords:** senescence, endothelial dysfunction, adipocytes, caloric restriction, oxidative stress

## Abstract

Cellular senescence is a state of permanent cell cycle arrest accompanied by metabolic activity and characteristic phenotypic changes. This process is crucial for developing age-related diseases, where excessive calorie intake accelerates metabolic dysfunction and aging. Overnutrition disturbs key metabolic pathways, including insulin/insulin-like growth factor signaling (IIS), the mammalian target of rapamycin (mTOR), and AMP-activated protein kinase. The dysregulation of these pathways contributes to insulin resistance, impaired autophagy, exacerbated oxidative stress, and mitochondrial dysfunction, further enhancing cellular senescence and systemic metabolic derangements. On the other hand, dysfunctional endothelial cells and adipocytes contribute to systemic inflammation, reduced nitric oxide production, and altered lipid metabolism. Numerous factors, including extracellular vesicles, mediate pathological communication between the vascular system and adipose tissue, amplifying metabolic imbalances. Meanwhile, caloric restriction (CR) emerges as a potent intervention to counteract overnutrition effects, improve mitochondrial function, reduce oxidative stress, and restore metabolic balance. CR modulates pathways such as IIS, mTOR, and sirtuins, enhancing glucose and lipid metabolism, reducing inflammation, and promoting autophagy. CR can extend the health span and mitigate age-related diseases by delaying cellular senescence and improving healthy endothelial–adipocyte interactions. This review highlights the crosstalk between endothelial cells and adipocytes, emphasizing CR potential in counteracting overnutrition-induced senescence and restoring vascular homeostasis.

## 1. Introduction

Cellular senescence is a complex process where cells cease to divide and are resistant to apoptosis [1]. Although senescent cells stop proliferating, they remain metabolically active and exhibit characteristic features, including increased cell size and the secretion of various factors [2]. Cellular senescence has emerged as a critical focus of research due to its pivotal role in the pathogenesis and progression of metabolic disorders, including obesity and type 2 diabetes (T2D). Central to this process is the intricate relationship between metabolism and aging, where excessive caloric intake emerges as a critical determinant driving senescence and age-related diseases [3,4].

Excessive nutrient intake, particularly from high-calorie diets, such as the Western diet—characterized by nutrient-poor foods such as fast foods, soft drinks, and highly processed foods—has been linked to obesity and conditions like metabolic syndrome, T2D, and cardiometabolic diseases [5,6]. Overnutrition affects key cellular metabolic pathways, including insulin/insulin-like growth factor signaling (IIS) [7,8], the mammalian target of rapamycin (mTOR) [9,10], and AMP-activated protein kinase (AMPK) [11,12]. These pathways play critical roles in regulating energy balance, and their dysregulation contributes to the progression of metabolic diseases and drives cellular senescence, further exacerbating age-related dysfunctions.

The chronic activation of IIS due to nutrient overload leads to insulin resistance and promotes cellular senescence by impairing glucose uptake and metabolism [8,13]. High nutrient intake activates the mammalian target of rapamycin complex 1 (mTORC1) signaling, which promotes cell growth and suppresses autophagy—a crucial process for clearing damaged organelles and proteins—thereby contributing to premature aging and the progression of metabolic diseases [10,14].

In contrast, AMPK—a sensor of nutrient stress, normally activated under conditions of energy deficiency—is suppressed by overnutrition, reducing fatty acid oxidation and insulin sensitivity [12]. This interruption promotes metabolic derangements and cellular damage and accelerates aging [7]. Targeting these pathways through caloric restriction (CR), exercise, or pharmacological agents has proven to be effective in restoring balance, improving insulin sensitivity, and promoting longevity [15]. Metabolism and aging are associated through mitochondrial function, crucial for energy production and central to aging [16,17]. Mitochondrial function declines with age and excessive caloric intake exacerbates this decline, accelerating cellular aging processes [18,19]. Progressive mitochondrial dysfunction increases the production of reactive oxygen species (ROS) that cause oxidative damage to cellular macromolecules and slow mitochondrial turnover [18,19].

The crosstalk between endothelial cells (ECs) and adipocytes is essential for metabolic regulation, particularly in the context of cardiometabolic diseases. Endothelial dysfunction (ED), driven by reduced nitric oxide (NO) production and oxidative stress in obesity, impairs vasodilation and blood flow [20]. Adipocytes secrete key mediators such as adiponectin, which enhances NO production and activates AMPK in ECs, promoting vasodilation and metabolic balance [21,22]. Additionally, extracellular vesicles (EVs) facilitate communication between ECs and adipocytes, regulating multiple processes that include lipid metabolism and autophagy [23,24,25].

When preparing this review, a literature search of PubMed, Scopus, Web of Science, and Google Scholar databases from 2001 to 31 December 2024 was conducted using the following medical subject heading terms: ‘obesity’ AND ‘senescence’ in combination with ‘caloric restriction’, ‘Western diet’, ‘metabolic syndrome’, ‘exosomes’, ‘extracellular vesicles’, ‘microRNA’, ‘lipotoxicity’, ‘type 2 diabetes mellitus’, ‘non-alcoholic fatty liver disease’, ‘inflammation’, ‘endothelial cells’, ‘adipocytes’, ‘adipose tissue’, and ‘overnutrition’.

This time frame was chosen as it represents a period of notable progress in the field. Instead, the historical milestones in CR research, shown in the figure in Section 5, highlight contributions from earlier periods. The search was restricted to English-language articles and reviews. The titles and abstracts of the results were screened, and full manuscripts that appeared to be relevant were reviewed. The reference lists of these papers were also examined to identify additional studies for inclusion in our survey.

The interplay between metabolic dysregulation, age-related diseases, and cellular senescence is a pivotal research area with profound implications for understanding disease progression and developing innovative therapeutic strategies. Although CR has been extensively studied for its health and longevity benefits, a comprehensive, up-to-date review of its impact on the crosstalk between adipose tissue and endothelial cells remains lacking.

This paper offers an in-depth review of recent advances on how metabolic dysregulation accelerates age-related diseases, drives cellular senescence, promotes chronic inflammation, and disrupts the interplay between adipose tissue and endothelial cells. In parallel, it highlights the importance of CR in extending the lifespan and delaying age-related diseases through several mechanisms that are linked to the improvement of mitochondrial function, reduction in oxidative stress, and promotion of autophagy, addressing its influence on the crosstalk between adipose tissue and endothelial cells [4,26,27,28].

## 2. An Overview of Cellular Senescence

Recent advances in molecular biology and genetics have provided valuable insights into the mechanisms of senescence. Twelve key hallmarks of aging have been proposed, including genomic instability, telomere attrition, epigenetic changes, loss of protein homeostasis, impaired macro-autophagy, deregulated nutrient sensing, mitochondrial dysfunction, replicative senescence, stem cell exhaustion, altered intercellular communication, chronic inflammation, and dysbiosis [2,14,29]. However, a complete understanding of the aging process remains elusive.

**Figure 1:**
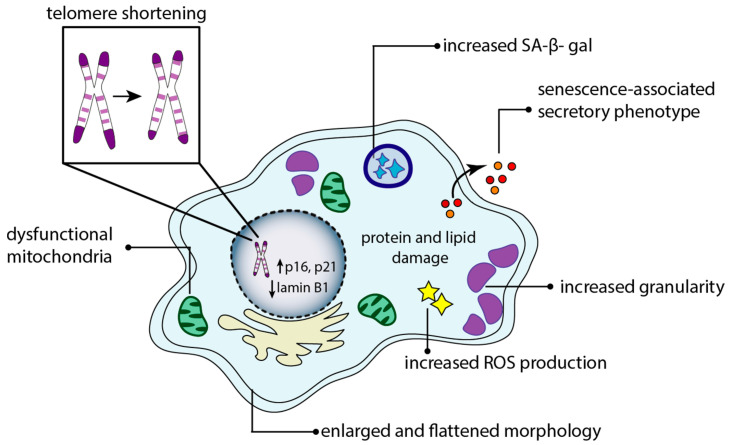
Hallmarks of a senescent cell. Senescent cells are defined by several key features. First, they exhibit a state of permanent cell cycle arrest, evidenced by p21, and/or p16 expression, and telomere shortening. Second is their morphology changes, becoming enlarged and flattened. Finally, they undergo multiple intracellular modifications, including mitochondrial dysfunction and the accumulation of reactive oxygen species (ROS) as well as damaged proteins and lipids at high levels. Senescent cells have a peculiar senescence-associated secretory phenotype (SASP) program, consisting of multiple cytokines and chemokines, and are positive for the senescence-associated beta-galactosidase (SA-β-gal) protein.

Today, cellular senescence is recognized as a fundamental mechanism of aging and can be triggered not only by natural aging (replicative senescence) but also by external stressors, such as oxidative stress, metabolic dysfunction, radiation, and genotoxic drugs (stress-induced premature senescence) [1,30]. Controlled cellular senescence is essential for maintaining tissue homeostasis. However, when this process is dysregulated, the excessive accumulation of senescent cells can drive chronic inflammation and tissue damage, playing a key role in the development of various age-related diseases [13]. The consequences of cellular senescence depend on the corresponding pathways that are activated. Stressors, such as oxidative stress, metabolic dysfunction, radiation, and genotoxic drugs, trigger various mechanisms such as DNA damage, telomere erosion, mitochondrial dysfunction, and oncogene activation [2,31]. The latter changes can result in the production of the senescence-associated secretory phenotype (SASP), which is characterized by the secretion of specific cytokines, chemokines, proteases, and growth factors [30,32]. SASP composition depends on the types of cells and tissues that are affected, as well as on the nature of the senescence-inducing stresses. Notably, Interleukin-6 (IL-6), IL-8, and Tumor Necrosis Factor-alpha (TNF-α) are considered as the most common components of the SASP [32,33] (Figure 1).

SASP factors not only reinforce senescence within the secreting cell (autocrine effect) but they can also induce cellular senescence in nearby non-senescent cells (paracrine effect), amplifying chronic inflammation and age-related disorders [34].

Morphologically, senescent cells exhibit changes such as increased size, flattened shapes, granulated cytoplasm, multinucleation, and DNA damage, which is detected by the increase in the phosphorylation of γH2AX, a marker of DNA double-strand breaks [35].

In senescent cells, the nuclear membrane becomes altered due to decreased lamin B1, a crucial regulator of chromatin structure [36]. Moreover, the lysosomal activity, detectable via SA-β-Gal staining, increases [37].

Key regulators of the senescent state include the tumor suppressor pathways p53 and retinoblastoma, which are activated in response to unresolved DNA damage. These pathways control cell cycle inhibitors such as p21 and p16, ensuring persistent cell cycle arrest [38,39]. Interestingly, the activity and level of p53 are crucial for determining whether a cell undergoes senescence or apoptosis, emphasizing the role of this specific protein in regulating the balance between cell cycle arrest and programmed cell death [40]. The activation of anti-apoptotic pathways, such as phosphatidylinositol 3-kinase (PI3K)/AKT signaling and B-cell lymphoma-2 (BCL-2)/BCL-XL pathways, allows senescent cells to evade apoptosis [30,32]. The identification of pathways that sustain the survival of senescent cells has paved the way for the development of senolytic drugs—compounds specifically designed to eliminate these cells [41]. These drugs operate by targeting vulnerabilities unique to senescent cells, inducing their apoptosis while sparing healthy cells [42]. Early senolytic agents, such as Dasatinib, a tyrosine kinase inhibitor, and Quercetin, a plant-derived flavonoid, have shown promising results demonstrating improvements in physical function, reductions in age-related pathologies, and extensions of the health span [43,44,45]. Dasatinib and Quercetin act on distinct subsets of senescent cells, underscoring the complexity and heterogeneity inherent in senescence biology. Cellular senescence is regulated by epigenetic mechanisms, including DNA methylation, histone modifications, and non-coding RNAs [46]. Epigenetic interference refers to heritable changes in gene expression, in the absence of changes in the DNA sequence [47]. During senescence, chromatin undergoes significant structural and functional changes [48]. DNA methylation consists of the covalent attachment of a methyl group to the C5 position of cytosine residues in CpG dinucleotide sequences, by methyltransferase enzymes, preventing gene transcription [49]. Its modifications include both global hypomethylation and focal hypermethylation at CpG-rich promoter regions [50,51,52]. DNA methylation is influenced by aging and environmental exposures [53,54]. A methylome-wide study of 718 individuals aged 25–72 years demonstrated a correlation between aging and increased methylation at CpG islands, shores, and shelves [55]. Several studies have highlighted DNA methylation as a valuable biomarker for measuring biological aging, distinguishing it from chronological age [53,56,57]. DNA methylation patterns, particularly those associated with epigenetic clocks, like Horvath’s and Hannum’s models [53,57], provide some insight into the biological aging process at the molecular level. These clocks are predictive of health outcomes, including the onset of age-related diseases, mortality risk, and overall health span.

On the other hand, histone post-translational modification may play an important role in the aging mechanism. Indeed, the basic unit of chromatin is the nucleosome, comprising 147 base pairs of DNA wrapped around a histone octamer containing H2A, H2B, H3, and H4 [58]. Histones undergo post-translational methylation and acetylation, which regulate gene expression by remodeling chromatin.

Deacetylation, through histone deacetylase, induces gene silencing while acetylation activates its transcription [59]. Acetylation neutralizes the positive charge of lysins, leading to DNA de-condensation, and facilitates transcription, often by enhancing transcription factor binding to target genes. Histone H3 lysine 9 can be acetylated or methylated, and both changes have been associated with aging [60].

Other epigenetic mechanisms of cellular senescence involve non-coding RNAs [61]. More than 90% of the human genome consists of genes that do not encode proteins. Despite this, these regions exhibit significant transcriptional activity, producing diverse non-coding RNAs, such as microRNAs (miRNAs), and long non-coding RNAs (lncRNAs), with essential regulatory roles in biological processes including senescence [1,36,62,63].

## 3. Aging of Adipose Tissue and Its Effect on Endothelial Cells: Mechanisms and Pathophysiological Consequences

### 3.1. Senescence in Adipose Tissue: Impact on Mitochondrial Function, Secretome, and Microvascular Interaction

With the global rise in obesity and T2D, the biology and physiology of adipose tissue have attracted considerable attention. In fact, the adipose tissue has long been recognized not only as a fat-storing tissue but also as an active metabolic and endocrine organ that plays a key role in regulating overall body function [64,65]. Accordingly, the adipose tissue plays a crucial role in maintaining lipid and glucose homeostasis, through the secretion of various adipokines, including adiponectin, leptin, resistin, and omentin [21,66].

The proper regulation of both lipogenesis and lipolysis is essential, as interferences in these processes due to obesity and T2D can severely impact adipose tissue metabolism [67].

White adipose tissue (WAT) is distributed throughout the body and is classified into two main typologies: subcutaneous adipose tissue (SAT), found beneath the skin, and visceral adipose tissue (VAT), which surrounds internal organs and is located mainly within the abdominal cavity [68]. SAT is typically more accessible for energy storage, while VAT is often associated with metabolic risk factors due to its proximity to vital organs, influencing metabolic health and contributing to various diseases, including cardiovascular conditions and insulin resistance [68]. Notably, perivascular adipose tissue (PVAT), owing to its close anatomical proximity to blood vessels, significantly influences vascular biology [69]. Therefore, the pathological impact of adipose tissue is influenced by its cellular composition, secretion profiles, and anatomical location within the human body.

The adipose tissue is composed of various cell types, including mature adipocytes of different sizes, adipose tissue-derived progenitor cells (APCs), microvascular ECs, and multiple immune cell types. Of note, this tissue undergoes significant alterations with age, and those modifications are mainly characterized by cellular senescence, immune senescence, and chronic inflammation, all of which contribute to poor metabolic health outcomes [29,70]. The size of mature adipocytes is commonly used as a key indicator for linking cellular functions to pathophysiological conditions. Studies in ethnically diverse populations have shown that enlarged mature SAT cells are linked to insulin resistance and may independently predict the onset of T2D, regardless of total body fat or body mass index [71,72]. Notably, fat cell hypertrophy has been shown to be associated with telomere shortening—an indicator of biological or premature aging—along with reduced adiponectin levels and increased oxidative stress in obese individuals with T2D [73,74,75].

Recent studies have shown that adipose tissue is considered the most vulnerable to aging, as it displays the earliest signs of cellular senescence in animal models [76]. In adipose tissue, cellular senescence impacts both APCs and mature adipocytes, impairing cell regeneration and resulting in diminished adipogenesis [74,77]. Senescent APCs exhibit high levels of p53 and p16, two markers of cell senescence, and produce SASP factors, such as activin A, which suppress adipogenesis in nearby non-senescent cells [78,79]. The inhibition of activin A in senescent cells has been shown to restore adipogenic capabilities in progenitor cells, suggesting a potential therapeutic target to counteract age-related metabolic decline [80,81].

The activation of p53 is central to the senescence process, inhibiting adipocyte differentiation and promoting inflammation, which exacerbates insulin resistance and metabolic dysfunction [82]. In individuals with hypertrophic obesity and T2D, senescent APCs accumulate, further impairing adipose tissue regenerative capacity [74,83]. Notably, senescent APCs exhibit reduced differentiation capacity into mature adipocytes, leading to an impaired browning process [83].

Senescence in mature adipose cells has been observed in both animal models and obese individuals [33,82,84]. These cells display DNA damage markers (such as γH2AX phosphorylation), express cell senescence-related genes (p53, GLB1, SERPINE-1), and secrete high amounts of SASP, contributing to chronic inflammation and insulin resistance [84]. Cell cycle regulators cyclin D1, p16, and β-GAL levels were progressively increased in mature adipocytes when lean, obese, and T2D individuals were compared [33]. In addition, in mature adipocytes, levels of β-GAL, p16, plasminogen activator inhibitor 1 (PAI-1, encoded by SERPINE-1), p53, and phosphorylated JNK1 were elevated in T2D individuals compared to lean individuals [33]. These changes were associated with a reduced expression of adipogenic markers, including peroxisome proliferator-activated receptor gamma (PPARγ) and GLUT4, as well as diminished insulin sensitivity, reflected by a decreased phosphorylation of AKT [33].

The secretome, composed of cytokines, chemokines, and growth factors, has an important role in amplifying oxidative stress, inducing metabolic dysfunction, and accelerating aging processes within the adipose tissue [2,78,85]. Immuno-senescence leads to the release of pro-inflammatory cytokines such as TNF-α, IL-1β, IL-6, and monocyte chemoattractant protein-1 (MCP-1) by WAT macrophages, which are also associated with senescent adipocytes [86,87]. The dynamic interplay between adipocytes and immune cells exacerbates the inflammation within adipose tissue, ultimately impacting systemic health [88]. Thus, cellular senescence is closely linked to inflammation and impaired metabolism in the adipose tissue of obese and T2D subjects [83].

Mitochondrial dysfunction is a key feature of cellular senescence, which is characterized by decreased respiratory capacity, increased ROS production, and alterations in mitochondrial dynamics [89]. Mitochondria are dynamic organelles whose functionality relies on a fine-tuned balance between their biogenesis, dynamic structural changes (fission and fusion), and turnover through mitophagy (a selective form of autophagy that removes damaged mitochondria) [90,91]. Different adipocyte subtypes—white, brown, and beige—exhibit distinct mitochondrial characteristics, affecting their roles in adipocyte differentiation, lipogenesis, and thermogenesis [92]. The conversion of white to brite adipocytes in humans depends on molecular, morphological, and functional mitochondrial changes, allowing brite/beige cells to perform thermogenesis [91]. Since mitochondria play a pivotal role in these activities, some studies have shown that aging in WAT adipocytes correlates with decreased mitochondrial efficiency, which is specifically characterized by a reduction in mitochondrial complex IV [93] and oxidative damage in aging WAT [93,94,95].

On the other hand, sirtuins, a family of NAD^+^-dependent protein deacetylases, regulate energy metabolism, DNA repair, and inflammation, playing pivotal roles in age-related diseases like diabetes [96]. In WAT, sirtuins regulate many pathophysiological processes, such as lipid mobilization, inflammation, fibrosis, the differentiation process, and browning [97,98,99,100]. SIRT1 activation in mouse adipocytes enhances fat mobilization and adiponectin production [99,101]. With aging, reduced SIRT1 expression leads to biotin accumulation in adipose tissue, perturbing energy metabolism via the biotinylation of carboxylases and histones [99]. The overexpression of SIRT1 alleviates aging-associated biotin accumulation and reduces the amount of biotinylated proteins, including acetyl CoA carboxylase, a major reservoir of biotin in adipose tissues [101]. Interestingly, chronic biotin supplementation eliminates adipose SIRT1-mediated beneficial effects on insulin sensitivity and lipid metabolism [101].

Meanwhile, microvascular endothelial cells (MVECs) derived from adipose tissue are also susceptible to cell senescence, especially in obese individuals. The microvascular endothelium regulates fatty acid transport and adipose tissue expansion in response to metabolic needs. During rapid adipose tissue expansion, as observed in obesity, the vascular network fails to grow adequately, leading to adipose tissue dysfunction [102,103].

A critical factor in adipose tissue function is the dynamic crosstalk between MVECs and adipose cells in vivo. This interaction plays a pivotal role in regulating lipid transport, ensuring the efficient delivery of fatty acids to adipose cells. Cellular senescence in MVECs impairs lipid transport and communication with adipose cells, contributing to adipose tissue dysfunction and the deposition of fat in ectopic sites, that is, outside the AT, which further worsens metabolic complications [103]. Additionally, MVECs contribute to the activation of PPARγ in APCs, which is essential for initiating and sustaining the adipogenic program, which drives the differentiation of APCs into mature adipocytes and maintains overall adipose tissue homeostasis [83]. Further, senescent MVECs in VAT exhibit a reduced expression of the PPARγ receptor, which is important for lipid metabolism and adipogenic regulation [102,103].

As discussed in the following paragraphs, this interplay underscores the importance of vascular–adipose communication in metabolic regulation and senescence. Senescent adipocytes can release EVs that negatively impact neighboring cells, thereby influencing metabolic health and reinforcing the cycle of cellular aging [104,105,106]. EVs can be classified into two main types, based on their size and biogenesis: large EVs, generated from the outward budding of the plasma membrane, and small EVs, originating from the fusion of intracellular multivesicular bodies with the plasma membrane before their release to the outside of the cell [107,108]. EVs carry various cellular components, including nucleic acids, lipids, and proteins, and play important roles in processes such as communication and waste management [25]. One of the first insights into EVs was their role in horizontal gene transfer, particularly RNA. EVs are enriched with miRNAs, leading to the hypothesis that EVs actively select RNA for intercellular transport [109,110,111].

### 3.2. Endothelial Cell Senescence: Impact on Vascular Remodeling, Glucose Metabolism, and Secretome

Early studies have shown that EC senescence happens in vivo and is detectable in various animal models and human tissues under multiple pathophysiological conditions [41,112]. Senescent ECs are triggered by aging, damage, or disease, and have a putative role in accelerating vascular diseases. For instance, balloon denudations in rabbit carotid arteries trigger EC senescence as marked by senescence-associated beta-galactosidase (SA-β-gal) staining [113]. Diabetic rat models also show increased endothelial cell senescence, particularly at 22 weeks of age, and the latter mechanism is related to the activation of the p53, p21, and p16 pathways [114]. Also, various studies have revealed senescent ECs in atherosclerotic plaques; subsequently, senescent cell clearance has been proposed as a promising therapeutic approach to mitigating the burden of atherosclerotic disease [115,116].

Concurrently, vascular aging involves a significant remodeling of the vessel wall that is characterized by lumen enlargement, thickening of both intimal and medial layers, and increased vascular stiffness [16,87]. These changes are coupled with increased endothelial permeability that is accompanied by a deterioration of the integrity of the vessel wall, all of which play critical roles in the aging process and the development of T2D [16,87].

Typically, ECs rely on glycolysis for most ATP production, with mitochondria serving primarily for biosynthesis and signaling rather than energy [117]. However, senescent ECs display reduced glycolysis and mitochondrial oxidative phosphorylation, potentially leading to altered energy sources like glutaminolysis [61]. Elevated glutamine consumption in senescent cells contributes to glutamate and lactate production but fails to sustain ATP levels, indicating a senescence-associated metabolic shift and increased cellular stress. It is worth mentioning that mitochondrial fission (splitting) increases in response to cellular stress, allowing the segregation of damaged parts for removal. In EC, aging promotes excessive mitochondrial fission due to oxidative stress, reduced fusion protein activity, and impaired mitophagy [19]. Consequently, damaged mitochondria accumulate, producing more ROS, triggering cellular dysfunction, and activating the SASP [19,118,119].

During cellular senescence, epigenetic alterations play a pivotal role in driving structural and functional changes in chromatin, impacting gene expression, and contributing to vascular aging [48]. For instance, in human ECs, transcription factors such as activating transcription factor 3 (a member of the AP-1 family) influence chromatin remodeling, altering gene expression and promoting senescence [120]. The hypermethylation of genes encoding endothelial nitric oxide synthase (eNOS) and extracellular superoxide dismutase (SOD3) leads to reduced endothelial protection, contributing to oxidative stress and atherosclerosis [121,122]. More specifically, it has been shown that Smyd3 induces histone H3 lysine 4 methylation, which increases Poly (ADP-Ribose) Polymerase Family Member 16 expression, leading to a subsequent rise in endoplasmic reticulum stress in response to angiotensin II and culminating in cellular senescence [123]. In turn, the histone acetyltransferase KAT7 promotes H3 lysine 14 acetylation, and enhances a cyclin-dependent kinase inhibitor, leading to a senescent phenotype [124].

Sirtuins play critical roles in counteracting vascular aging by regulating EC function and mitigating senescence by targeting SIRT1. Interestingly, EC-specific SIRT1 knockout mice exhibit an age-related decline in blood flow and endurance [125]. Conversely, the overexpression of endothelial SIRT1 or NAD^+^ supplementation restores capillary density and blood flow [125]. This protective action occurs partly by inhibiting PAI-1, a marker and mediator of cellular senescence [126]. Similarly, the deletion of SIRT6 accelerates vascular aging while SIRT6 overexpression prevents EC senescence and mitigates hypertension by inducing GATA5 expression, which is mediated by the deacetylation of histone H3 lysine 9 [127]. Also, in progeria mice, the EC-specific restoration of SIRT7 activity reduces vascular aging and extends the lifespan [128].

Basisty et al. [129] highlighted that the composition of the SASP is dynamic and varied depending on cell type and the stressor-inducing senescence, reflecting its adaptability in aging and disease situations. For example, in ECs, the SASP components include pro-inflammatory cytokines (IL-6, IL-8), growth factors, extracellular proteases (MMPs), and pro-thrombotic factors (PAI-1) [130]. The latter cocktail mediates multiple pathological effects, including impaired NO bioavailability and insulin signaling [42]. Given the large area covered by ECs, which is estimated to be between 4000 and 7000 m^2^, the impact of endothelial SASP on systemic physiology is expected to be extremely profound [85]. The accumulation of senescent ECs is thought to promote inflammaging that has significant implications for metabolic health, particularly in the context of diabetes [86]. Evidence indicates that ex vivo samples of the endothelium that are isolated from aged and T2D individuals display the persistent activation of nuclear factor-kappa B (NF-κB), a transcription factor regulator of inflammatory responses [131]. Chronic NF-κB activation in ECs drives their inflammatory state, providing a potential mechanism through which ED contributes to the pathophysiology of aging and metabolic disorders [132].

Senescent ECs release EVs, which play a critical role in vascular dysfunction. These vesicles contribute to the progression of vascular diseases by promoting senescence in vascular smooth muscle cells [133]. More specifically, these senescent ECs’ specific EVs contain regulatory miRNAs and lncRNAs, which are involved in modulating gene expression and cellular processes, further exacerbating vascular aging and disease [133]. For instance, under normal physiological conditions, endothelial cell-derived EVs promote vascular repair by transferring miR-126 to recipient cells, enhancing tissue regeneration. However, under hyperglycemic conditions, EVs exhibit a significant reduction in miR-126 levels, leading to impaired repair capacity both in vitro and in vivo. This shift in EV function underscores the detrimental impact of diabetes on vascular repair mechanisms, potentially contributing to long-term vascular dysfunction and disease progression [134,135,136]. Endothelin-1 (ET-1), a vasoconstrictor primarily produced by endothelial cells, triggers the release of endothelial EVs with altered phenotypes and cargo [137]. ET-1-induced EVs negatively affect endothelial health by promoting inflammation, inhibiting eNOS expression, and reducing NO production. This occurs through the release of inflammatory cytokines such as IL-6 and IL-8, while the concomitant reduction in miR-146a and miR-181b expression further contributes to ED and vascular damage [137].

## 4. Excessive Caloric Intake

### 4.1. Impact on Intracellular Metabolic Pathways and Cell Senescence

**Figure 2:**
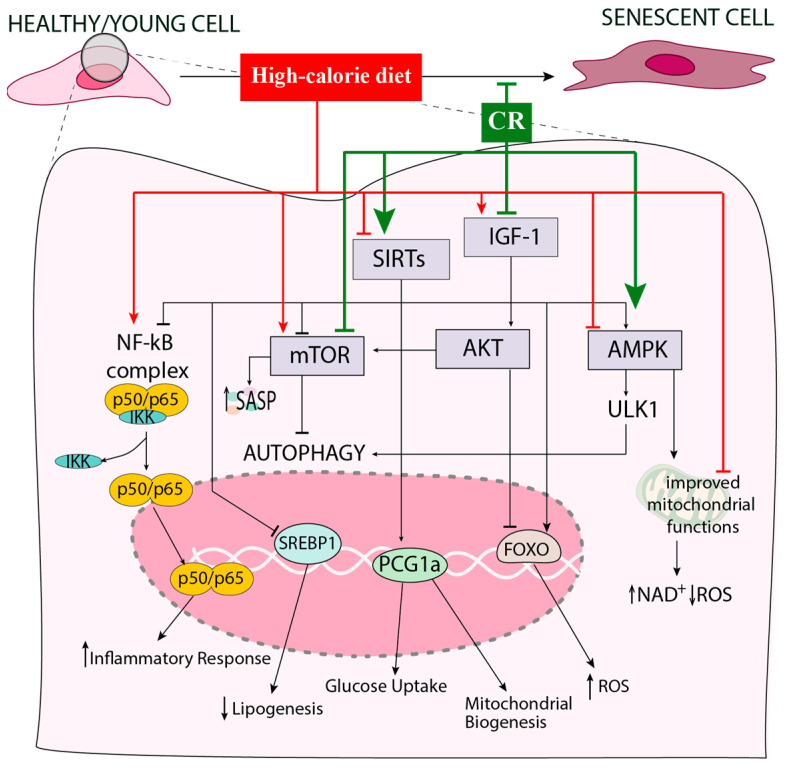
Biochemical pathways involved in endothelial senescence are affected by dietary factors, such as a high-calorie diet and caloric restriction. A high-calorie diet suppresses two critical pathways, essential for maintaining endothelial function: NAD^+^-dependent protein deacetylase sirtuins (SIRTs) and AMP-activated protein kinase (AMPK). Caloric restriction (CR) increases NAD⁺ levels, activating SIRTs. CR reduces ATP levels, leading to an increased AMP/ATP ratio, which activates AMPK and stimulates the autophagic process via ULK1. Additionally, a high-calorie diet activates the mammalian target of rapamycin (mTOR) and insulin/insulin-like growth factor 1 (IGF-1) pathways, which are associated with increased inflammatory responses, the reactive oxygen species (ROS) stress-induced translocation of NF-κB to the nucleus, senescence-associated secretory phenotype (SASP) induction, and dysregulated autophagy. In contrast, caloric restriction exerts protective effects by modulating key intracellular pathways involved in cellular senescence, including AMPK, mTOR signaling, and the IGF-1 axis. These changes promote enhanced DNA repair, lipid metabolism, autophagy, and resistance to oxidative stress.

Excessive caloric intake initiates a cascade of detrimental effects on the cells, disturbing multiple biological pathways that maintain metabolic and cellular homeostasis [3,6,11,14] (Figure 2). This condition leads to cellular stress, oxidative damage, and chronic inflammation, all accelerating cellular senescence [16,74,84].

Among the pathways heavily impacted by overnutrition is the IIS pathway, which is a crucial regulator of metabolism, growth, and longevity [8,138,139]. It mediates cellular responses to insulin and IGF-1, promoting glucose uptake, protein synthesis, and cell growth. Investigations across various species, from worms to mice, have suggested that the downregulation of the GH and IGF-1 signaling pathways can promote lifespan extension [140,141,142]. Conversely, chronic insulin resistance impairs glucose metabolism, leading to increased blood glucose levels and the development of T2D, which are associated with accelerated aging and increased morbidity [6,143,144]. Furthermore, the hyperactivation of the IIS pathway promotes cellular senescence through the upregulation of senescence-associated genes and proteins, such as vascular cell adhesion molecule 1 (VCAM-1), cyclin-dependent kinases, the nuclear NF-κB p65 subunit, and SA-β-Gal, thus contributing to the decline in tissue function [46,145].

Another key player that is influenced by a high-calorie diet is the mammalian target of rapamycin (mTOR) [68]. The mTOR pathway plays a central role in regulating cell growth, proliferation, and metabolism in response to nutrient availability [9,10,146]. mTOR is a member of the phosphatidylinositol 3-kinase-related kinase family of protein kinases and is differentially associated with other proteins, acting as a core component of two distinct protein complexes, mTORC1 and mTORC2, which regulate various cellular processes [10]. The activation of mTORC1, a nutrient-sensing pathway and master regulator of multiple metabolic signaling pathways, promotes protein synthesis, nucleotide synthesis, lipogenesis, and glycolysis, and inhibits autophagy and lysosome biogenesis [14,147,148]. Alternatively, mTORC2 regulates cell survival/glucose metabolism and cytoskeletal remodeling [10]. The overactivation of mTORC1 is linked to excessive nutrient intake, leading to increased protein synthesis and decreased autophagy [149].

AMPK, a heterotrimeric serine/threonine protein kinase, is the enzyme responsible for sensing nutrients and is thus deeply involved in obesity [11,14]. AMPK plays a critical role in regulating adipose tissue metabolism [150,151]. It promotes catabolic processes that generate ATP and inhibits anabolic processes that consume energy [150]. Excessive caloric intake leads to decreased AMPK activation, resulting in reduced fatty acid oxidation and augmented fat storage [11]. This metabolic dysregulation contributes to obesity and insulin resistance, further exacerbating the aging process [12,152].

Finally, excessive caloric intake has been reported to target the mammalian sirtuins (SIRT1–7), which are involved in various physiological and pathological processes [153,154]. These processes include the regulation of the cell cycle, mitochondrial biogenesis and function, glucose and lipid metabolism, insulin sensitivity, inflammatory responses, and overall energy homeostasis [96,128,153,155,156].

In conclusion, excessive caloric intake alters vital metabolic pathways, particularly by the overactivation of both IIS and mTOR pathways and suppressing AMPK and sirtuin activity [7]. These interferences impair glucose metabolism, reduce autophagy, and promote insulin resistance, cellular senescence, and chronic inflammation, all key drivers of aging and age-related diseases [7,157]. Conversely, as detailed in the subsequent sections, CR has been shown to mitigate these effects by enhancing autophagy, activating AMPK/sirtuins, and inhibiting mTOR and IIS, thereby improving mitochondrial function and metabolic health.

### 4.2. Impact on Adipose Tissue and Endothelial Cell Communication

**Figure 3:**
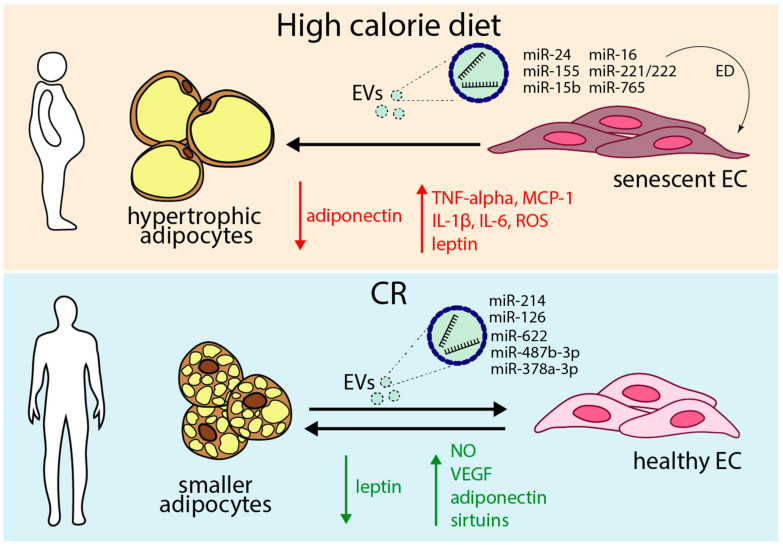
Adipocyte–endothelial cell crosstalk. Cell-to-cell communication between adipocytes and endothelial cells (ECs) is crucial in maintaining the homeostasis of both tissues and the whole organism. The main actors in this talk are several cytokines that are differentially produced by the two cell types, as well as micro-RNA-carrying extracellular vesicles (EVs). While obesity is linked to an increase in the production of pro-inflammatory cytokines (e.g., TNF-alpha, IL-1β, IL-6) and micro-RNAs (miR) that are involved in boosting endothelial dysfunction (ED) and senescence, caloric restriction (CR) is conversely involved in the upregulation of specific miRs and anti-inflammatory mediators (e.g., NO, VEGF) that are responsible for preserving a healthy EC phenotype.

Over the years, significant attention has been focused on how excess adiposity contributes to vascular dysfunction. The cross-communication between ECs and adipocytes has gradually emerged as a crucial element in metabolic regulation, especially in the context of cardiometabolic diseases (Figure 3) [158,159].

WAT becomes dysfunctional in obesity, creating a pro-inflammatory, hyperlipidemic, and insulin-resistant environment that ultimately contributes to the development of metabolic and vascular complications [92,150,158].

In a healthy state, the crosstalk between adipocytes and ECs supports metabolic homeostasis and vascular integrity [25]. However, with aging and in conditions such as obesity, the crosstalk between these cells can become dysfunctional, leading to ED and increased inflammation [160].

Early studies focused on ED, which contributes significantly to insulin resistance and related metabolic complications [161]. The first key finding was that obesity-induced ED is characterized by an impairment in flow-mediated vasodilation, which is mainly due to a dysfunction in eNOS, resulting in a loss of NO production, which is a principal vasodilatory and anti-inflammatory agent [20,162].

These changes were linked to reduced blood flow in WAT in response to stimuli such as food intake, particularly in obese individuals compared to lean subjects [163]. Notably, the transgenic overexpression of eNOS in animal models protected against diet-induced obesity and insulin resistance, further underscoring the vital role of ECs in maintaining metabolic homeostasis [164]. ED can shift the vascular endothelium to a pro-inflammatory, pro-thrombotic, and pro-atherogenic phenotype [159]. This transition results in leukocyte adhesion, and platelet activation, along with an impaired production of endothelial NO, decreased synthesis of endothelium-derived hyperpolarizing factors, and increased levels of vasoconstrictors such as angiotensin II and prostaglandin H2 [165].

The mechanism by which overnutrition impairs NO production has been partially elucidated. The eNOS interacts with caveolins—a family of integral membrane proteins essential for the formation and function of caveolae—which regulate its activity, thereby modulating NO production and influencing vascular functions [166]. In the saphenous arteries of diet-induced obese rats, the levels of caveolin-1 monomers and oligomeric complexes of caveolin-1 and -2 were reported to increase in membrane-enriched samples, potentially impairing eNOS dissociation from caveolin-1 and compromising NO-mediated vasodilation; nonetheless, obesity was not shown to affect eNOS or caveolin-3 expression in these rats [166].

Compellingly, a recent study identified the T-type calcium channel Cav3.1 as a regulator of endothelial function in obesity [167]. Mice lacking Cav3.1 are resistant to high-fat diet-induced obesity and exhibit improved endothelial function [167]. It was shown that the Cav3 channel interacts with eNOS in ECs, although the exact mechanisms remain unclear [168]. Similarly, during obesity, many miRNAs regulate pathways involved in cell cycle arrest, inflammation, and DNA damage response. For instance, miR-24, miR-155, miR-15b, miR-16, miR-221/222, and miR-765 levels are overexpressed in the adipose tissue [36,169,170,171,172]. These miRNAs are responsible for eNOS inhibition via AKT and caveolin signaling, leading to ED and atherosclerosis [36]. Preclinical and clinical evidence suggests that leptin is a crucial player in obesity-associated ED and hypertension especially in females, due to the increase in the association between leptin and aldosterone [173,174]. Recent studies have shown that females develop a heightened renin–angiotensin–aldosterone system activation compared to males [175]. In fact, the leptin-induced aldosterone production, combined with the increased expression of endothelial mineralocorticoid receptors in females, highlights the importance of the leptin–mineralocorticoid receptor axis in the obesity-associated, leptin-induced vascular endothelial impairment in women [174,175]. Finally, obesity-induced hyperleptinemia promotes atherosclerosis by driving selective leptin resistance, preserving harmful vascular effects while dampening metabolic benefits [176].

Adiponectin is a vital anti-inflammatory adipokine that plays a crucial role in metabolic regulation, significantly influencing insulin sensitivity and anti-inflammatory processes [21,22]. Lower levels of adiponectin are associated with obesity, T2D, and various metabolic disorders [177,178]. Adiponectin enhances insulin sensitivity in skeletal muscle and suppresses hepatic glucose production [177,179]. Moreover, it stimulates NO production in ECs via its receptors, AdipoR1 and AdipoR2, whose activation triggers AMPK, leading to vasodilation [180]. Thus, adiponectin is essential for maintaining metabolic health and vascular function [178,181]. Moreover, adiponectin stimulates the endothelial secretion of EVs [25]. Recently, Blandin et al. [182] identified the enrichment of oligomeric forms of adiponectin in small EVs, with the adipokine mainly localized close to the EV surface due to the nonspecific adsorption of its soluble form. Adiponectin-enriched sEVs exhibit insulin-sensitizing effects in vitro, engaging standard adiponectin receptors. Also, in high-fat-fed mice, adoptive transfer of these sEVs prevents weight gain, improves insulin resistance, and reduces tissue inflammation, with clear benefits observed in the adipose tissue and liver [182].

It is worth noting that circulating EV levels are elevated in obese individuals compared to lean, healthy subjects [183,184]. Moreover, plasma EV levels correlate with body mass index and the homeostatic model assessment of insulin resistance, suggesting a potential role for EVs in the pathogenesis of T2D [183,184]. Notably, blood injections of small EVs derived from obese AT into lean mice induced whole-body insulin resistance compared to lean AT-derived small EVs [185], suggesting that the metabolic effects of EVs rely on the intercellular transfer of EV cargos.

Some studies have demonstrated that adipocyte-derived EVs account for a large portion of circulating miRNAs in humans and mice [23,36]. Mice with adipocyte-specific dicer knockouts—an enzyme essential for miRNA processing—exhibited significantly lower plasma miRNA levels in EVs, indicating that adipocytes significantly contribute to circulating miRNAs [23]. These miRNAs have functional roles in the post-transcriptional control of the gene expression of multiple genes that play a key role in metabolic processes such as glucose tolerance and liver function [23]. For instance, adipose tissue-derived miRNAs, such as miR-21, mediate obesity-induced EC dysfunction by modulating the expression of genes involved in eNOS, SIRT1, ROS production, autophagy, and endoplasmic reticulum stress [36]. Additionally, the PVAT was reported to secrete various miRNAs, including miR-221-3p, which is notably enriched in obese PVAT; on this same note, recent studies have shown that miR-221-3p contributes to ED by promoting structural changes in blood vessels [186].

Environmental factors, such as hypoxia, have also been shown to affect EV secretion [187,188]. Some studies have shown that WAT expansion in obese individuals is associated with reduced oxygen tension [189]. This contributes to WAT inflammation and fibrosis, worsening systemic insulin resistance [189]. Under low-oxygen conditions, EVs released from adipocytes impair insulin-stimulated glucose uptake, suggesting a link between hypoxia and metabolic dysfunction [188]. Proteomic analyses have revealed that EVs from hypoxic adipocytes contain more lipogenesis-related proteins, including fatty acid synthase, pyruvate kinase, glyceraldehyde-3-phosphate dehydrogenase, and ATP synthase subunit beta, compared to EVs from normoxic adipocytes [24].

Endothelium-derived EVs transfer the caveolin-1 protein to adipocytes, a process regulated by feeding and fasting [25]. Caveolin-1 is involved in fatty acid storage into adipocytes: during fasting, caveolin-1 transfer increases, while it is decreased in high-fat-fed or genetically obese mice [25]. Taken together, these studies suggest that EV-mediated communication between ECs and adipocytes is critical in metabolic regulation, particularly in response to dietary changes.

In this particular context, ceramides were shown to influence EV production, suggesting that EV secretion might serve as a mechanism to reduce cellular stress in ECs [190]. Adiponectin promotes EV secretion in ECs, reducing ceramide accumulation and further highlighting the importance of EVs in cellular waste management and communication [24].

T-cadherin, another key protein in adiponectin signaling, is a glycosylphosphatidylinositol-anchored receptor expressed in various tissues, including the heart, skeletal muscle, and endothelium [191]. Mice lacking T-cadherin show elevated plasma adiponectin levels, suggesting that T-cadherin regulates systemic adiponectin levels [191]. Additionally, adiponectin binding to T-cadherin appears to influence EV production, potentially through T-cadherin oligomerization, further enhancing the adiponectin role in intercellular communication [192].

Beyond their role in communication, EVs also participate in cellular waste management, a process that is similar to secretory autophagy and that helps cells eliminate harmful materials [193]. Blocking EV secretion leads to the accumulation of damaged DNA and the activation of stress response mechanisms in the cell [194]. Autophagy, a process closely related to EV secretion, is critical for the function of both adipocytes and ECs. Mice with adipocyte-specific knockouts of autophagy-related genes, such as Atg3 and Atg16L, develop inflammation in adipose tissue even in the absence of obesity [195]. These mice also exhibit insulin resistance, underscoring the importance of autophagy in maintaining metabolic health [195]. Similarly, autophagy plays a key role in ECs, where it helps regulate overall vascular functions [196].

Obesity-induced systemic or local inflammation and insulin resistance transform the adipose tissue secretome from an anti-inflammatory to a pro-inflammatory state [182]. This shift is characterized by hypertrophic adipocytes that secrete higher levels of pro-inflammatory adipokines like TNF-α, IL-6, IL-1β, leptin, and MCP-1, which promote low-grade inflammation and oxidative stress, particularly in VAT [21,197]. These factors contribute to vascular dysfunction and senescence by reducing protective adiponectin levels and promoting a pro-inflammatory environment. Overall, the interplay between obesity, inflammation, and altered adipose tissue secretions is crucial in the progression of vascular complications.

Obese adipocytes, like senescent adipocytes, are characterized by functional alterations such as reduced adiponectin levels and increased inflammatory signaling [198]. These changes promote ED by creating a pro-inflammatory environment, exacerbating vascular damage.

The dysfunctional interaction between ECs and adipocytes creates a vicious cycle of metabolic and vascular deterioration [69]. Inflamed and stressed adipocytes exacerbate endothelial damage, leading to further adipocyte dysfunction, while ED impairs nutrient delivery and waste removal in adipose tissue [173,193,199].

## 5. CR Delays Aging and Reduces Age-Related Disease Vulnerability

Aging is an inevitable process, but several studies have demonstrated that its progression can be modulated by factors such as diet and lifestyle.

Dietary interventions have become essential tools for promoting health, preventing chronic diseases, and potentially extending the lifespan. These strategies encompass various approaches, including intermittent fasting [200], time-restricted feeding [201], ketogenic diets [202,203,204], and the modulation of specific macronutrients [205,206], such as protein or amino acid restriction. Intermittent fasting alternates eating and fasting periods, while time-restricted feeding confines food intake to specific windows, both of which improve metabolic flexibility, enhance insulin sensitivity, and support circadian rhythm optimization [200,201]. Ketogenic diets, characterized by high fat and low carbohydrate intake, induce ketosis, promoting fat metabolism and showing potential in managing obesity, neurological disorders, and metabolic conditions [204]. Restricting protein and amino acids, such as methionine, has garnered attention for its influence on pathways like mTOR, which play critical roles in aging and metabolic health [207,208]. Despite their differing mechanisms, these interventions share the common objective of optimizing metabolic functions and alleviating the burden of age-related and lifestyle-associated diseases. Among these, CR remains the most extensively studied and well-documented dietary intervention [209,210,211].

**Figure 4:**
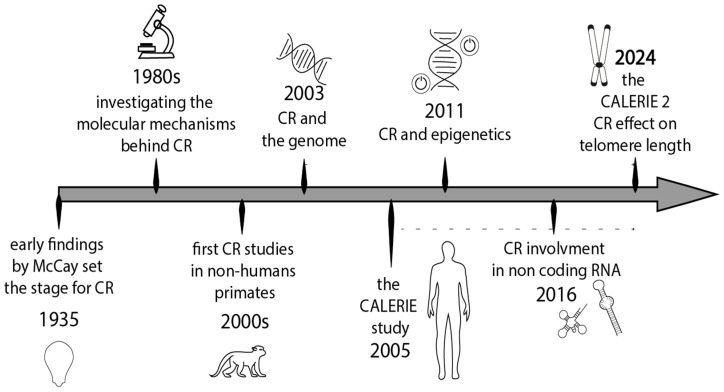
A timeline of key milestones in caloric restriction research. This timeline outlines key milestones in caloric restriction (CR) research, beginning in 1935 with McCay’s demonstration that CR extends the lifespan in rodents. By the 1980s, the focus shifted to uncovering the molecular mechanisms underlying CR’s effects [217,218,219]. In the following decades, studies have expanded further to nonhuman primates [220], and the understanding of genomic implications [221] and epigenetic effects [222]. An important milestone was the CALERIE (The Comprehensive Assessment of Long-Term Effects of Reducing Intake of Energy—CALERIE™), the first clinical trial [223] that investigated CR effects in humans. In 2016, the involvement of CR in long non-coding RNAs [224] was initiated. Finally, the effect of CR on telomere shortening was studied in the CALERIE 2 trial [225].

In 1935, McCay and colleagues discovered that long-term CR initiated after weaning could extend the lifespan of rats [212]. Since then, several studies in rodents have shown that CR enhances overall metabolism, reduces age-related pathophysiological changes, and promotes longevity [211,213,214,215,216]. A timeline of the significant dates and discoveries that have shaped the understanding of CR is illustrated in Figure 4.

CR is defined as the reduction (20–60%) in typical calorie consumption, below energy requirements, while maintaining optimal nutrition [226]. Meanwhile, it was previously reported that people who follow plant-based diets with low sugar and animal protein intake tend to live longer compared to those who consume a high-calorie, low-fiber Western diet [227].

There is compelling evidence that CR leads to long-lasting epigenetic effects that modulate the expression of age-related genes [222,228]. CR influences three key types of epigenetic alterations: DNA and histone methylation, and histone acetylation [49]. CR can increase the activity of DNA methyl transferases 3, which is responsible for maintaining the methylation of genes involved in inflammation and diabetes, which are usually demethylated with aging [229].

Studies in nonhuman primates and clinical trials have demonstrated that CR-induced weight loss can modify the methylation of genes linked to inflammation, such as TNF-α, as well as genes related to weight control and insulin secretion [230]. Mice subjected to 40% energy restriction starting at 12 weeks of age demonstrated a longer lifespan and improved hepatic DNA methylation, with the hypermethylation of genes related to fatty acid and ketone body metabolism [228].

Thus, the potential of CR to enhance the lifespan and delay aging has drawn much interest in the last decade.

In the following sections, we explore the potential mechanisms by which CR can modulate multiple pathways and processes that regulate diverse functions of the adipose tissue and endothelium, by highlighting the changes that CR inflicts at the level of their secretomes and intercellular communications.

### 5.1. CR Improves the Health and Longevity of Adipose Tissue

Due to their central involvement in energy storage, metabolism, and signaling, adipocytes play a pivotal role in mediating the body’s response to CR. As mentioned above, aged adipose tissue tends to become dysfunctional, accumulating hypertrophic (enlarged) adipocytes, and contributing to a pro-inflammatory environment [21]. Omics studies show that CR modulates aging processes and delays age-related diseases by reducing oxidative stress, improving metabolism, suppressing adiposity and insulin resistance, and reprograming the lipidome and metabolome [231]. Proteomic analyses reveal that CR improves glucose and lipid metabolism and minimizes oxidative damage [232]. A key aspect of aging is the loss of regenerative capacity in the adipose tissue. Adipose-derived stem cells are crucial for tissue regeneration, but aging diminishes their stemness and regeneration potential [233,234]. CR holds promise as a potential strategy to rejuvenate the stemness of aged stem cells [233].

A transcriptomic analysis of adipose tissue revealed that CR suppresses the transcription and activity of genes involved in the inflammatory response, such as those related to the NF-κB signaling pathway [235].

CR increases adiponectin levels, which improves insulin sensitivity and exhibits anti-inflammatory effects, simultaneously decreasing leptin levels, which can otherwise contribute to inflammation and insulin resistance when present in excess [21,236]. Studies on WAT morphology have revealed that CR reduces adipocyte size and alters gene expression profiles, particularly in genes related to fatty acid biosynthesis [237]. CR upregulates Sterol regulatory element-binding proteins (SREBP-1c), enhancing fatty acid biosynthesis specifically in WAT, and suppresses inflammation through GH/IGF-1-independent mechanisms [237].

Other organelles affected by aging are the mitochondria. In aged adipocytes, they become less efficient, leading to an increased production of ROS and a decline in metabolic flexibility [93,238]. CR helps reverse this trend by stimulating mitochondrial biogenesis and reducing oxidative stress. The mitochondrial efficiency enhances and improves the metabolic flexibility of adipocytes, enabling them to switch between carbohydrate and fat metabolism more effectively [92,239]. CR enhances mitochondrial biogenesis in WAT by upregulating PPARγ coactivator-1α (PGC-1α), a master regulator of mitochondrial function [240]. This process involves the activation of nuclear respiratory factors (NRF-1 and NRF-2), which promote the expression of mitochondria-related genes, including mitochondrial transcription factor A, which is essential for mitochondrial DNA replication and transcription [241]. Additionally, CR has been shown to activate fibroblast growth factor 21 signaling in WAT, which further enhances PGC-1α expression and promotes mitochondrial biogenesis [242].

CR depletes cellular energy, promoting a catabolic state, altering nutrient-sensing pathways, and increasing NAD^+^ production [243,244]. The NAD^+^/NADH ratio that is high during low-energy states is pivotal for cellular energetics, as NAD^+^ activates SIRTs, a family of NAD^+^-dependent deacetylases [245,246,247]. As mentioned earlier, SIRTs function as intracellular nutrient sensors, coupling energy status with post-translational modifications such as protein and histone deacetylation, ADP-ribosylation, and mitochondrial protein acylation [155,248,249]. These modifications regulate nuclear gene transcription, metabolism, and immunity, mediating epigenetic changes critical for cellular adaptation.

Elevated NAD^+^ during CR enhances SIRT activity, integrating metabolic and transcriptional responses [246,247].

CR modulates SIRT activity through pathways that regulate cellular energy and redox balance. CR increases NAD⁺ levels by activating NAD⁺ biosynthetic enzymes like nicotinamide phospho-ribosyl-transferase (NAMPT) and inhibiting NAD⁺-depleting enzymes such as PARP and CD38 [250,251]. The elevated NAD⁺/NADH ratio activates SIRTs, which depend on NAD⁺ to function. Additionally, CR induces energy stress (low ATP levels), activating AMPK, which in turn promotes NAMPT expression, further enhancing NAD⁺ biosynthesis and indirectly stimulating SIRT activity [250].

SIRT1 is a key responder to CR-induced energy changes, facilitating transcriptional regulation and genome integrity during low-energy states. Primarily located in the nucleus, SIRT1 deacetylates histones (H1, H3, H4) and transcription factors, maintaining a heterochromatic environment and genomic stability. SIRT1 appears to sense nutrient availability by responding to increased levels of NAD^+^ during CR, which subsequently induces widespread alterations in the acetylation of cytosolic and nuclear proteins, including histones [252,253,254].

CR enhances the expression and activity of SIRT1 in various tissues, including adipose tissue, further contributing to the inhibition of NF-κB and the reduction in inflammation [155,252].

SIRT3 in the mitochondrial matrix regulates energy metabolism, stress resilience, and ROS detoxification [255]. These modifications boost oxidative stress response and fatty acid β-oxidation, supporting energy efficiency and antioxidant defenses under low-energy conditions.

CR increases the expression of SIRT3, COX4 (a component of the mitochondrial respiratory chain), and Tom20 (a component of mitochondrial protein import machinery) in WAT, but not in other tissues, in a SREB-1c-dependent manner [256].

SIRT6 enhances insulin secretion, promotes fatty acid β-oxidation, and inhibits adipogenesis through the deacetylation of key targets [153,257]. It regulates protein secretion, including TNF-α, and reduces inflammation by deacetylating NF-κB regions. These functions, tied to NAD^+^ availability, contribute to anti-inflammatory and anti-aging effects observed during CR [258,259].

Increased autophagy is associated with improved cellular health and longevity, as it can mitigate age-related diseases and inflammation [260]. CR also enhances autophagy, helping to remove damaged cells, responsible for the acceleration of aging processes [260,261]. Autophagy is regulated by several pathways, including mTOR, S6K, and Akt, which inhibit autophagic processes, and AMPK and SIRT1, which promote autophagy [146,253]. CR activates lipophagy, a specialized form of autophagy where adipocytes break down stored lipids in lysosomes [239,262]. This process is critical for maintaining cellular homeostasis and preventing the accumulation of dysfunctional lipid molecules that can exacerbate inflammation, oxidative stress, and senescence.

In addition, CR promotes the browning of WAT, transforming it into metabolically active beige fat. Beige adipocytes enhance thermogenesis, burning energy to generate heat, which helps prevent visceral fat accumulation; supports energy balance; and reduces the risk of metabolic dysfunction during aging [92].

On the other hand, CR positively influences the immune environment within adipose tissue [68]. It decreases the presence of pro-inflammatory macrophages while enhancing anti-inflammatory immune cell populations, thereby improving the immune profile of adipose tissue [210]. This transition toward an anti-inflammatory environment under CR significantly reduces insulin resistance and preserves metabolic health during aging [210]. In obese mouse models, a CR of 30% for two months has been shown to significantly reduce cytokine and chemokine levels, such as IL-6, IL-2, IL-1Rβ, MCP-1, and CXCL16 (key components of the SASP), in the adipose tissue [263]. In the liver, even mild CR was shown to significantly suppress the expression of pro-inflammatory and lipogenic genes, including MCP-1, SREBPs, and PPARs [264]. PPARα and PPARγ, which are essential in lipid and carbohydrate metabolism, decline with age but are restored by CR [264]. These findings indicate that CR effectively mitigates inflammation, which is linked to pathological conditions such as chronic inflammation, insulin resistance, and reduced energy metabolism [265]. This has long-term implications for preventing diseases such as T2D and cardiovascular disease, both of which are strongly associated with adipose tissue inflammation [68].

In summary, CR can extend the lifespan and improve metabolic health, by reducing inflammation and lipophagy, enhancing mitochondrial function, and maintaining the regenerative capacity of adipose tissue.

### 5.2. CR Is a Promising Strategy to Combat Vascular Aging and Improve Endothelial Function

Several studies highlighted the significant impact of CR on vascular health and longevity, revealing its complex interplay with various cellular pathways.

Mice fed a diet that met their caloric needs but was lower in protein and branched-chain fatty acids showed reduced adiposity, increased metabolic rates, and longer lifespans [206]. The authors attributed these benefits not just to CR but to a decreased activation of the mechanistic target of rapamycin complex 1 (mTORC1) by amino acids and fatty acids. Throughout aging, mTORC1 activity increases, which correlates with eNOS uncoupling and the overproduction of superoxide anions (O₂⁻), both of which are associated with ED [266]. Interestingly, studies have shown that treating senescent ECs with rapamycin (an mTOR inhibitor) can reverse eNOS uncoupling and reduce oxidative stress [266]. Similar improvements in endothelial function have been observed in older mice subjected to a CR diet, highlighting the potential role of mTORC1 in mediating the vascular benefits of CR [267]. Indeed, CR has been shown to inhibit mTOR signaling, thereby promoting autophagy and enhancing cellular health [146,235].

CR also modulates EC function through other stress-induced cellular pathways, such as AMPK and SIRT1, by enhancing their expression [161,253]. CR reduces intracellular ATP levels, leading to an increased AMP/ATP ratio, which activates AMPK and stimulates the autophagic process via ULK1 [268,269,270]. Some studies have demonstrated that CR primarily activates AMPK, rather than altering its expression [7,15,253]. CR-induced AMPK activation enhances mitochondrial function and promotes cellular longevity [7,15,253]. AMPK activation stimulates NO synthesis, which helps maintain vascular function and prevent arterial stiffening [271]. AMPK activation, in turn, triggers the transcription of FOXO3 and Nrf2, resulting in sustained antioxidant effects [246]. The role of SIRTs in vascular aging, along with the effects of CR on SIRT activity and expression, has been extensively studied [132,246,253,272].

By deacetylating and activating transcription factors such as FOXO, SIRT1 enhances autophagic flux, thus promoting the clearance of damaged proteins and organelles that accumulate with age [132,246,253,272]. Therefore, one of the mechanisms through which CR can slow down vascular aging and improve endothelial function is through increased autophagy [132,253].

The role of SIRTs in vascular aging extends beyond their regulation of autophagy and metabolism. SIRT1 functions as a histone deacetylase, meaning it can influence gene expression through epigenetic modifications. Epigenetic regulation is increasingly recognized as a key determinant of aging, and CR has been shown to enhance SIRT activity, thereby promoting chromatin remodeling and maintaining genome stability [248,254]. By modifying the epigenetic landscape, CR can delay the onset of senescence and extend the health span of ECs, further supporting its potential use as a remedial intervention for vascular aging [28,248,254].

Another major factor contributing to vascular aging is the stiffening of the extracellular matrix, primarily due to the breakdown of elastin and collagen by matrix metalloproteinases (MMPs) and the accumulation of advanced glycation end-products (AGEs) [273,274]. In particular, MMP-9 plays a significant role in elastin degradation, while AGEs promote chronic inflammation by interacting with their receptors on ECs [275]. These processes lead to increased arterial stiffness, a hallmark of vascular aging and a major risk factor for cardiovascular diseases [276]. CR has been shown to reduce MMP-9 activity and inhibit the formation of AGEs, thus reducing extracellular matrix stiffening and the ensuing vascular damage [276]. In conclusion, CR enhances autophagy, reduces inflammation, and preserves endothelial function, by modulating key metabolic and stress-responsive pathways such as mTORC1, AMPK, and SIRT1.

### 5.3. CR Modifies Adipocyte and Endothelial Secretory Profiles and Improves Their Crosstalk

This section explores the molecular and physiological mechanisms through which CR modulates adipose–endothelial communication, highlighting its effects on adipocyte and endothelial secretory profiles including the SASP, adipokine, EV, and miRNA [277].

Age-associated endothelial senescent cells exhibit heightened SASP that develops a pro-inflammatory milieu detrimental to neighboring cells [278]. CR may improve metabolic function in part by reducing cellular senescence and the pro-inflammatory SASP. In a recent 18-week human randomized controlled trial, 31 participants (middle-aged/older adults) on CR lost 10.8 kg, significantly reducing endothelial-related SASP biomarkers, including soluble ICAM-1 [279].

CR reduces leptin levels, which may alleviate leptin-induced ED and improve vascular health [280]. Interestingly, a study showed that serum leptin was reduced by 31% following CR in humans and this reduction was associated with a decrease in the activation of blood CD4 + CD25 + T-lymphocytes [281].

Similarly, CR has also been shown to increase circulating levels of adiponectin, thereby promoting endothelial wellbeing [282,283]. Adiponectin is known to exert anti-inflammatory effects by inhibiting the expression of some adhesion molecules like ICAM-1 and VCAM-1 on ECs [284]. Studies in both rodent models and humans have demonstrated that CR upregulates adiponectin expression in adipocytes, subsequently enhancing NO production in ECs, and improving the vasodilatation [282]. This shift in the adipokine profile under CR conditions is critical for maintaining endothelial homeostasis. Adequate vascularization ensures the efficient delivery of oxygen and nutrients to adipocytes and facilitates the removal of metabolic waste products.

One of the key regulators of angiogenesis is the vascular endothelial growth factor, which is secreted by adipocytes and ECs in response to hypoxic conditions [68,285]. CR has been shown to increase the expression of pro-angiogenic factors in adipose tissue, thereby enhancing vascular density and improving tissue oxygenation [285]. Improved vascularization improves adipocyte function and attenuates hypoxia-induced inflammation and oxidative stress, promoting a rejuvenated microenvironment that benefits both adipocytes and ECs [285,286].

Also, CR improves insulin sensitivity in adipocytes by reducing circulating levels of IGF-1, insulin, and glucose, thereby inhibiting PI3K/Akt signaling, leading to a decrease in lipolysis and circulating FFAs [287,288,289]. This reduction in FFA levels protects ECs from lipid-induced stress and inflammation, thereby improving vascular health [288]. CR-increased insulin sensitivity in ECs enhances NO production and contributes to maintaining the vascular tone [236,267]. As mentioned above, WAT-derived EVs play an important role in the intercellular and inter-organ crosstalk [106,111,185,277,290]. Thus, preventing or reverting the EV accumulation may protect against multiple obesity-related metabolic dysregulation and ED [291]. In obese humans, circulating levels of EVs, enriched with perilipin A (an adipocyte marker), decreased by 35% after 3 months of CR [291]. Jaimes et al. [187] demonstrated that in mice undergoing 21 days of 30% CR, the EVs isolated exhibited greater protective properties against hypoxia/reperfusion injury compared to those from ad libitum-fed mice. In the same study, EVs from human blood samples collected before and after a 72 h water-only fast showed different EV cargos [187]. The composition of the cargo underwent a huge shift reflected in a shift from EVs containing high levels of miR-622, miR-487b-3p, and miR-378a-3p to EVs incorporating lower amounts of miR-760, miR-98-5p, and miR-31-5p.

Other studies have also reported some changes in miRNA profiles under altered calorie intake in several tissues [292,293,294]. For instance, aging in mice is associated with a reduction in the expression of numerous miRNAs in adipose tissue, a change largely prevented by CR. This phenomenon is primarily linked to a decrease in components of the miRNA processing machinery, particularly the enzyme dicer, which is essential for miRNA maturation [293]. Therefore, the regulation of miRNA processing in adipose tissue during aging is a phenomenon that is mediated by metabolic signals and that integrates the harmful effects of stress with the beneficial effects of CR during the process of aging [293].

The levels of miR-34a increase with aging, promoting cellular senescence [295]. A mouse study revealed that CR improves liver metabolism by downregulating miR-34a, which enhances fatty acid oxidation, promotes cholesterol efflux, and inhibits cellular senescence [296]. Offspring of obese mothers showed adipose tissue stress mediated by miR-126 [297], a crucial regulator of physiological angiogenesis in endothelial progenitor cells. A study explored how exercise and diet improve microvascular endothelial dysfunction in obese adolescents [298]. Over six weeks, the intervention group showed significant improvements in body measurements, glycolipid metabolism, the reactive hyperemia index, and NO/ET-1 levels, with decreased miR-126 levels. Changes in miR-126 correlated with BMI, the reactive hyperemia index, and NO/ET-1, suggesting its role in vascular function. Inside of the cell, CR improves mitochondrial protein homeostasis via the miRNA-induced activation of mitochondrial translation. CR-induced miRNAs like miR-122 enhance mtDNA-encoded protein production, triggering the mitochondrial unfolded protein response, which preserves mitochondrial function and supports longevity [292].

Another study revealed the pivotal role of lncRNA KCNQ1OT1 in cellular senescence and CR [299]. KCNQ1OT1 knockdown triggers senescence markers and ROS generation via CK2α downregulation, regulated by the KCNQ1OT1-miR-760-CK2α axis [299]. In ECs, there are several miRNAs involved in endothelial functionality and wellbeing that have been extensively studied. Among all, miR-126 is altered after CR and is involved in reduced VCAM-1 expression, and reduced inflammation and oxidation [300,301]. In addition, miR-126 is involved in the regulation of Nrf2, a transcription factor that is responsible for cellular response to oxidative stress, metabolic control, oxidized phospholipid signaling, and the repair and degradation of damaged macromolecules [302]. CR preserves youth in cerebral–microvascular ECs by maintaining Nrf2 activity, reducing oxidative stress, and modulating multiple corresponding miRNAs [302]. CR prevents miR-144-induced Nrf2 suppression, reduces inflammation, and supports endothelial homeostasis, counteracting vascular aging and dysfunction [303].

### 5.4. Translational Potential and Limitations of CR in Human Health

CR has demonstrated remarkable benefits in preclinical studies across various species. These include an enhanced lifespan, improved metabolic health, and protection against age-related diseases such as neurodegeneration, cardiovascular disorders, and cancer. Translating these findings into human health holds immense potential but also presents significant challenges.

Emerging evidence from human clinical trial studies suggests that CR can improve markers of metabolic health, such as insulin sensitivity, lipid profiles, and inflammation levels, while potentially influencing biomarkers of aging. Trials like CALERIE (Comprehensive Assessment of the Long-term Effects of Reducing Intake of Energy) have shown that moderate CR can reduce oxidative stress and improve overall health markers in non-obese adults [223]. A 24-month CR trial in healthy adults showed preferential adipose tissue loss, especially VAT, compared to muscle and organ tissues. CR significantly affected VAT, intermuscular fat, muscle, and liver volumes [304].

A 12-week intensive lifestyle intervention combining a low-carb, high-protein calorie-restricted diet, exercise, and personalized counseling significantly improved liver steatosis and metabolic outcomes in overweight/obese Chinese patients with NAFLD, compared to a standard intervention. Intensive lifestyle intervention also reduced BMI and improved various metabolic markers, with notable benefits in patients with hypertension [305]. Another study investigated the effects of a CR diet on oxidative/antioxidative status in NAFLD patients. While CR led to significant weight loss, reductions in BMI, and improved liver enzymes, it did not significantly alter oxidative stress markers or FGF-21 levels, suggesting that long-term interventions with greater weight loss are needed [306].

Despite all the promising results and significant advancements, integrating CR into public health strategies requires a deeper understanding of its effects on diverse populations, its compatibility with different lifestyles, and the potential risks of malnutrition or muscle loss in vulnerable groups. A 2-year CR trial in non-obese adults resulted in significant weight, fat, and fat-free mass loss, alongside reduced bone mineral density at key fracture sites [307]. Increased markers of bone turnover and reduced physical activity levels highlight the potential impact of CR on bone health, which warrants further study.

The application of CR in humans must account for variations in individual physiology, cultural dietary practices, and long-term adherence. Personalized nutrition, considering the genetic background, microbiome, and individual metabolic profiles, could enhance intervention effectiveness. These approaches highlight the importance of tailored dietary strategies to address the growing burden of obesity, NAFLD, and its associated metabolic complications, offering a comprehensive path for prevention and treatment [308]. A recent DRIFT study [309], a 52-week randomized trial, compared daily CR and intermittent fasting for the treatment of long-term obesity, with the primary outcomes focusing on sustainable, evidence-based obesity management. Although daily CR is the standard approach, its adherence often decreases over time, leading to modest weight loss and frequent weight regain. Intermittent fasting, which involves the energy restriction of more than 60% for 2–3 days per week or every other day, offers an alternative strategy, but has not been adequately studied in long-term studies.

## 6. Conclusions

Obesity is driven by inflammation, oxidative stress, and impaired immune functions and it can induce metabolic dysregulations similar to the ones seen in aging. Chronic inflammation in obesity promotes adipose tissue enlargement and exhibits many hallmarks of aging, such as mitochondrial dysfunction, cellular senescence, and reduced autophagy. Excessive ROS that are generated during this inflammatory state exacerbate oxidative stress, impair mitochondrial DNA integrity, and disrupt cellular homeostasis. Obesity also reduces autophagic flux, leading to the accumulation of dysfunctional organelles and misfolded proteins, mirroring aging-related cellular decline. On the other hand, the chronic activation of DNA damage response and p53 induces senescence and the activation of SASP. These processes accelerate biological aging, evidenced by telomere shortening and faster epigenetic clock progression, contributing to age-associated diseases.

CR can counteract these effects by restoring autophagy, and metabolic balance, and reversing aberrant epigenetic modifications associated with aging. Therefore, the epigenome may be an interesting target in this context, as it is responsible for many crucial physiological mechanisms that are related to aging, and thus its modulation may be considered very promising for conferring healthy and prolonged life. Advancing our understanding of epigenetics in human aging and longevity necessitates exploring how environmental factors influence these mechanisms, particularly in CR-mediated longevity regulation. CR has been considered for several years as a powerful intervention to improve health and longevity, as it targets key mechanisms of aging through various physiological processes. Its benefits are particularly evident for adipose tissue and vascular health, influencing key cellular and molecular pathways, mitigating inflammation and metabolic dysfunction, and the adverse effects of aging. By modulating pathways such as mTORC1, AMPK, and SIRT1, CR reduces endothelial oxidative stress, enhances autophagy, and increases the production of NO, a key mediator for maintaining vascular tone and preventing arterial stiffening. CR also improves endothelial function through its anti-inflammatory actions, mitigating the SASP, and restoring vascular integrity. Finally, CR improves adipose–endothelial crosstalk by modulating secretory profiles, including adipokines, EVs, and miRNAs, regulating mitochondrial function and inflammation.

Studying the potential synergistic effects of combining CR with senolytic drugs may offer new insights into reducing cellular damage and improving tissue function.

Exploring alternative, less invasive dietary interventions, such as intermittent fasting, may provide additional avenues to prevent senescence and promote healthy aging without the challenges associated with long-term CR. Moreover, integrating CR into public health strategies requires a deeper understanding of its effects on diverse populations, its compatibility with different lifestyles, and the potential risks of malnutrition or muscle loss in vulnerable groups. Precision nutrition approaches, exploiting genetic, epigenetic, and microbiome knowledge, could further optimize the impact of CR while minimizing negative effects.

This review includes preclinical studies conducted on various animal models and with dietary interventions ranging from 10% to 40% caloric reduction. The variability also extends to the duration of treatments, which ranged from 3 weeks to 7 months in animal studies and from 3 months to 2 years in clinical studies. This significant heterogeneity limits the comparability of the findings across studies.

In conclusion, CR translational potential in human health is vast, offering a promising strategy to delay aging and prevent chronic diseases. Ongoing research, combined with personalized and culturally adaptable interventions, will be essential to fully realize its potential benefits for public health.
